# Dose-dependence of protection from systemic reactions to venom immunotherapy by omalizumab

**DOI:** 10.1186/s12948-016-0051-2

**Published:** 2016-10-24

**Authors:** Elisa Boni, Cristoforo Incorvaia, Marina Mauro

**Affiliations:** 1 Allergy Unit, Sant’Anna Hospital, ASST Lariana, Via Napoleona 60, 22100 Como, Italy; 2 Allergy/Cardiac and Pulmonary Rehabilitation ASST G. Pini/CTO, Via Bignami 1, 20126 Milan, Italy

**Keywords:** Venom immunotherapy, Honeybee, Systemic reactions, Omalizumab

## Abstract

**Background:**

Systemic reactions (SR) to venom immunotherapy (VIT) are rare but may occur, with a rate significantly higher for honeybee than for vespid VIT. In patients with repeated SRs to VIT it is difficult to reach the maintenance dose of venom and pre-treatment with omalizumab is indicated, as shown by some studies reporting its preventative capacity, when antihistamines and corticosteroids are ineffective.

**Case presentation:**

We present the case of a 47 years old woman allergic to bee venom who experienced two severe SRs after bee stings and several SRs to VIT with bee venom. Pre-treatment with antihistamines and corticosteroids as well as omalizumab at doses up to 300 mg was unsuccessful, while an omalizumab dose of 450 mg finally achieved in our patient the protection from SRs to VIT with 200 mcg of bee venom.

**Conclusions:**

The search of the dose of omalizumab able to protect a patient with repeated SRs to VIT may be demanding, but this search is warranted by the need to provide to this kind of patient, by an adequate VIT, the protection from potentially life-threatening reactions.

## Background

Venom immunotherapy (VIT) is generally safe and, differently from injective immunotherapy with inhalant allergens, no fatal reaction to treatment has been reported [1]. Still, systemic reactions (SR) may occur, with a rate significantly higher for honeybee than for vespid VIT. In fact, a systematic review defined a rate of SRs of 25.1 % for honeybee VIT and 5.8 % for vespid VIT [2]. In patients with repeated SRs it is difficult to reach the maintenance dose of venom, usually corresponding to 100 mcg [1]. Mild to moderate SRs may be averted by pre-treatment with antihistamines [3], while for severe SRs pre-treatment with omalizumab is indicated, as shown by some studies reporting its preventative capacity [4–6]. However, a negative study was published [7]. We describe the case of a patient with repeated SRs to honeybee VIT who initially was apparently not responsive to the omalizumab treatment but achieved the complete prevention of SRs by dose increase.

## Case presentation

The patient is a woman exposed to honeybee stings because her father is a beekeeper. At the age of 22 years she experienced a SR of grade 4 severity according to Mueller [8] after a single bee sting. Honeybee venom hypersensitivity was then diagnosed by skin tests and VIT for bee venom was started. However, the treatment was withdrawn early, due to repeated SRs to VIT. No other stings until the age of 47 years when the patient had a further SR (again grade 4 according to Mueller) after a bee sting. Patient’s clinical features are reported in Table 1. According to clinical history, no additional allergy neither other medical conditions were present. In 2013, VIT for bee venom was then scheduled by honeybee venom from Stallergenes (Antony, France) but already during the build-up phase, at the dose of 10 mcg of venom, a SR with angioedema of the glottis, cough, itching of hands and feet occurred, requiring epinephrine administration for resolution of the symptoms (Table 2). Premedication with terfenadine 180 mg twice a day in the three days before VIT was attempted but anaphylaxis occurred again at the dose of 10 mcg and administration of epinephrine was again necessary. Therefore, VIT for bee was once more planned using premedication with terfenadine and anti-IgE for preventing SRs. Omalizumab 300 mg was administered twice with a 14 day interval during the build-up phase of VIT with a modified rush schedule at weekly interval (Table 3). However, when reaching the dose of 10 mcg the patient had cough and dysphagia. Changing the premedication to omalizumab plus intravenous hydrocortisone 500 mg, intravenous ranitidine 50 mg and cetirizine 10 mg/os, a maintenance dose of 200 mcg of bee venom was reached in 11 weeks and well tolerated in the following months. This suggested to step down omalizumab to 150 mg every 2 weeks and using oral premedication with prednisone 25 mg, rupatadine 10 mg and ranitidine 150 mg. VIT and omalizumab administrations were set on different days. However, when omalizumab was reduced to 150 mg once a month a SR requiring epinephrine occurred. Therefore, the dose of omalizumab was doubled to 300 mg once a month along with the oral premedication with the usual drugs letting the patient tolerating the monthly dose of 200 mcg of bee venom. However, seven months later, the same premedication regimen was not able to prevent a new SR to VIT. Finally, when increasing the dose of omalizumab to 450 mg monthly, 2 days before VIT, preceded by oral premedication with prednisone, rupatadine and ranitidine 12 and 2 h before VIT, the patient no longer suffered from SRs over the last 14 months and is still under regular treatment.

**Table 1:** Patient’s clinical features at first visit

Age at first visit in our clinic	47 years old
Sex	Female
Concomitant allergies	None
Concomitant diseases	None
Previous systemic reactions of grade IV Muller	Yes
Number of previous attempts of VIT with HB venom withdrawn for repeated systemic reactions during build-up phase	3
Skin test	Prick test HB venom: 20 mm (hystamine: 10 mm)
Total IgE	51 kU/l
s-IgE HB	20.3 U/ml
s-IgE Api m 1	7.93 U/ml
s-IgE Api m 10*	0.00 U/ml
s-IgE CCD	0.00 U/ml
Basal tryptase	2.4 ng/ml
Mastocytosis in bone marrow (biopsy performed)**	Absent
KIT mutation**	Absent

* performed in 2015; ** performed in 2016

**Table 2:** Previous attempts of buildup phase with HB venom

Week no.	Premedication	HB venom cumulative dose (mcg)	Adverse Reactions
1		4.11	None
2		10	Ocular itching
3		5	Anaphylaxis
5	Terfenadine	4.11	None
6	Terfenadine	10	None
7	Terfenadine	10	Anaphylaxis

**Table 3:** Build up phase with administration of omalizumab

Week no.	Premedication	Omalizumab (mg)	HB venom cumulative dose (mcg)	Adverse reactions
1		150		
3		300		
4	Terfenadine		4.11	None
5	Terfenadine	300	10	Cough and dysphagia
6	Hydrocortisone Ranitidine Cetirizine		20	None
7	Hydrocortisone Ranitidine Cetirizine	300	30	Ocular and palmar itching
8		300		
9	Hydrocortisone Ranitidine Cetirizine		45	None
10	Hydrocortisone Ranitidine Cetirizine	300	60	None
11	Hydrocortisone Ranitidine Cetirizine		80	None
12		300		
13	Hydrocortisone Ranitidine Cetirizine		100	None
14	Hydrocortisone Ranitidine Cetirizine	300	130	None
15	Hydrocortisone Ranitidine Cetirizine		170	None
16	Hydrocortisone Ranitidine Cetirizine	300	200	None

## Conclusions

VIT is a highly effective treatment but not all patients are protected from SRs by the usual maintenance dose of 100 mcg. Rueff et al. demonstrated that in all patients not completely protected from stings a protective dose may be individuated, that in rare cases may be as high as 400 mcg [9].

The case we report shows that also the search of the dose of omalizumab able to protect a patient with repeated SRs to VIT may be demanding, but this pursuit is warranted by the need to provide to this kind of patient, by an adequate VIT, the protection from potentially life-threatening reactions. In previous reports, the minimal effective dose of omalizumab to protect from systemic reactions to VIT was 150 mg [10], thus the search of the protective dose should start from 150 mg, with increase to 300 mg and, possibly, to 450 mg in case of incomplete protection. The most appropriate combination therapy including also corticosteroids and antihistamines is not yet established and needs be investigated.

## Abbreviations

SR: systemic reaction
VIT: venom immunotherapy
mcg: micrograms
mg: milligrams
